# Mobile Phone–Based Smoking-Cessation Intervention for Patients Undergoing Elective Surgery: Protocol for a Randomized Controlled Trial

**DOI:** 10.2196/12511

**Published:** 2019-03-26

**Authors:** Marcus Bendtsen, Catharina Linderoth, Preben Bendtsen

**Affiliations:** 1 Division of Community Medicine Department of Medical and Health Sciences Linköping University Linköping Sweden; 2 Department of Medical Specialists Motala Hospital Motala Sweden

**Keywords:** smoking cessation, mobile phone–based interventions, SMS, mHealth interventions

## Abstract

**Background:**

Several large studies have shown that the risk of cardiovascular, respiratory, and wound-healing complications (including death) within 30 days of surgery is greater for smokers than for nonsmokers. However, there is evidence that even short-term perioperative smoking cessation may reduce postoperative morbidity. Over the past few years, it has become more evident that short message service (SMS)–based interventions can help individuals quit smoking.

**Objective:**

The overall aim of this project is to fill the knowledge gap on whether an SMS-based smoking-cessation intervention can be effective in helping patients stop smoking perioperatively. The aim of this trial is to evaluate the effectiveness of an SMS-based intervention on smoking behavior of patients undergoing elective surgery.

**Methods:**

A two-arm parallel-group randomized controlled trial will be conducted at 20 surgical departments in southeast Sweden. Smokers undergoing elective surgery who own a mobile phone will be included. Power calculations indicate that it will be necessary to randomize 434 participants. One group will be given access to a novel 12-week SMS program, which includes daily SMS messages with behavior change–enforcing text content and hyperlinks to interactive modules, while the other group will not be given access to the intervention. Both groups will have access to the surgical departments’ current routine for smoking cessation prior to surgery. Primary outcome measures, prolonged abstinence, and point prevalence of smoking cessation will be measured through questionnaires at 3, 6, and 12 months after randomization. Logistic regression models adjusted using baseline characteristics will be explored to identify potential effects of the intervention.

**Results:**

Recruitment started in late October 2018 and is expected to last for a maximum of 30 months. The first results are expected to be available approximately 3 months after the final date of recruitment.

**Conclusions:**

Owing to the structural problems and scarcity of time and resources, patients at most Swedish surgical departments are simply instructed to quit smoking, and perhaps, referred to a primary health care clinic. An SMS-based smoking-cessation aid can be effective in helping individuals quit smoking and is a very simple and time-efficient tool for surgical departments to use.

**Trial Registration:**

ISRCTN Registry ISRCTN33869008; http://www.isrctn.com/ISRCTN33869008

**International Registered Report Identifier (IRRID):**

PRR1-10.2196/12511

## Introduction

### Background and Rationale

Smoking is responsible for more than 60 diseases and is the single most influential preventable factor for disease and premature mortality [1]. In Sweden, tobacco is associated with 9.6% of the total disease burden [2]. Around 6400 people die every year in Sweden due to smoking. The previously recorded decline in the number of smokers in Sweden has started to level out, and in 2016, the proportion of smokers among both women and men was 9% [3]. Among individuals aged 45-64 years, the proportion of daily smokers was higher than that for any other age group—13% for women and 9% for men. Including occasional smokers, these proportions increase to 16% for women and 13% for men [3].

Studies show that approximately 65% of all smokers want to quit, and approximately 50% make at least one quit attempt each year, but only 10% seek or gain access to evidence-based supportive measures [4]. In addition to the addictive nature of smoking, the cost of treatment and time commitment make cessation a challenge [5,6].

Over 70 years ago, The Lancet published an article describing increased surgical complications after operation for patients who were smokers [7]. Since then, several large studies have shown that the risk of cardiovascular, respiratory, and wound-healing complications (including death) within 30 days of surgery is greater for smokers than for nonsmokers [8,9]. However, there is evidence that even short-term perioperative smoking cessation may reduce postoperative morbidity [5,10].

The rationale behind smoking cessation before surgery is that the short-term harmful effects of carbon monoxide and nicotine in the blood disappear 24-48 hours after smoking cessation. The harmful effects of carbon monoxide are mediated by a reduction in the availability of oxygen to the tissues by 3%-12% and associated with an increased risk of cardiac arrhythmias [10]. Nicotine stimulates the surgical stress response and increases the blood pressure, pulse rate, and systemic vascular resistance, thereby increasing the workload of the heart [10]. As anesthesia and surgery cause an increased strain on cardiac and circulatory functions, an existing oxygen imbalance can be worsened in patients who smoke, potentially resulting in hypoxemia in vital organs. Smoking also induces mucus production, which might impede the clearance of mucus, leading to an increased risk of postoperative pulmonary infections in combination with a reduced immune function associated with smoking [11].

The optimal timing and intensity of smoking cessation before surgery to reduce postoperative pulmonary complications remain unclear, but the period before a planned surgery might still be seen as a window of opportunity for smoking interventions [8,10,12]. A multicenter randomized control trial where smoking cessation was instituted after operation for acute fracture showed a decreased risk of postoperative complications in the intervention group, indicating that even cessation after surgery can be beneficial [13]. In contrast, findings reported in a Cochrane review [10], based on indirect comparisons and evidence from two small trials, showed that interventions beginning 4-8 weeks before surgery and including weekly counseling were most likely to have a significant impact on complications and long-term smoking cessation.

### Previous Research and Findings

Over the past few years, it has become more evident that short message service (SMS)–based interventions can help individuals quit smoking. In a Cochrane review from 2016 [14], it was indicated that long-term quit rates increased by 67% among those who were given access to SMS-based smoking-cessation interventions compared to those who were not. These findings were supported by a second meta-analysis conducted the same year, which found that abstinence increased by 63% with the use of SMS-based interventions [15].

Our research group has previously investigated the use of an SMS-based intervention (NEXit) for smoking cessation among university students in Sweden [16-18]. The intervention was evaluated using a two-arm, parallel-group randomized controlled trial including 1590 participants between the ages of 21 and 30 years. With respect to 8-week prolonged abstinence, a statistically significant difference was found between the intervention group (25.9%) and control group (14.6%). Differences in the 4-week point prevalence between the groups were also statistically significant (20.6% versus 14.2%). The intervention has now been adapted to a younger age group and is currently in trial among high-school students in Sweden [19].

There is, however, no research in Sweden or elsewhere on the use of SMS-based interventions for smoking cessation prior to surgery. Given the success of the approach in other contexts and the importance of smoking cessation prior to surgery, it is valuable to determine if such an approach could work in this context.

### Aims and Hypotheses

There is strong evidence that short-term smoking cessation before surgery can reduce postoperative complications and morbidity. There are, however, several structural problems in the Swedish health care system concerning the organization of smoking cessation for patients waiting for surgery. The overall aim of this project is to fill the knowledge gap on whether an SMS-based smoking-cessation intervention can be effective in helping patients stop smoking preoperatively. This trial protocol includes a two-arm parallel-group randomized controlled trial that aims to evaluate the effectiveness of the SMS-based intervention on smoking behavior as a tool in addition to the current routine treatment and to measure potential mediators of the intervention on smoking cessation.

There are two hypotheses in this study: (1) Smoking outcome measures will differ between the two groups at 3, 6, and 12 months after randomization. The 3-month period will be the primary time point. (2) Importance of smoking cessation and self-efficacy will mediate the effects of the intervention on smoking outcomes at 3 months. The same measures at 3 months will mediate the effect of the intervention at 6 months (and 6 months on 12 months).

## Methods

### Intervention Content

#### Rationale

Previous cessation interventions have not been able to establish clear evidence of which components or techniques are most essential to include for smoking cessation [20-22]. In the absence of a clear theory as the basis of the intervention, the content of the text messages in the proposed intervention was based on our previous smoking-cessation research concerning university students, key elements from other internet- and SMS-based interventions, official manuals about smoking cessation, and books from cessation experts [14,16-18,23-32]. The length of the intervention of 12 weeks was set to conform with previous interventions, recommendations, and our own previous experience [23,26,28,31-37].

The messages were developed with the intention to encourage participants to quit smoking. The messages included information about the consequences of smoking, how to quit and stay smoke free, tasks to perform (such as getting rid of ashtrays and cigarettes), coping strategies to deal with cravings, motivational messages, and how to avoid smoking triggers. Content that specifically focused on the benefits of smoking cessation prior to the trial was added. One of the initial messages also contains a hyperlink to a video where a surgeon gives a motivational speech and explains why it is important to quit smoking prior to surgery.

#### Intervention

The proposed intervention consists of 130 messages that are sent via SMS to the participant’s mobile phone over a 12-week period. Two to four messages will be sent per day during the first few weeks, which will be reduced to two per day during the middle part of the intervention and further reduced to one message per day during the latter part of the intervention. Some of the messages include hyperlinks that take the participants to interactive modules that they should engage with. There are a total of nine such modules, and throughout the intervention period, participants will be reminded of their responses to modules they have completed in the past.

Briefly, the nine interactive modules that users engage with throughout the intervention are as follows:

1. A set of tips and tasks, of which participants choose five that suit them.
2. A set of reasons to quit smoking, of which the participants choose five (or enter their own reason).
3. A series of questions that result in a plan for how to deal with certain situations in which the temptation to smoke is increased, such as “When I have had my dinner, I will have a piece of fruit” or “While I am waiting for the bus, I will listen to music.”
4. A set of information boxes that relate to withdrawal symptoms.
5. A set of tips that participants choose, or enter on their own, on what to do when craving cigarettes.
6. A set of information boxes with information about what happens to the human body after smoking cessation.
7. A set of information boxes with information about good habits that can replace the smoking habit.
8. A series of questions that lead to suggestions of physical activities that the participant might want to try in order to improve his/her health further and relieve abstinence.
9. A pros and cons list created by the participant for smoking cessation.

In summary, the intervention is based on previous research and expert knowledge and has been designed to suit the specific context of the current situation of the participant—a situation in which they must quit smoking preoperatively.

### Design

**Figure 1:**
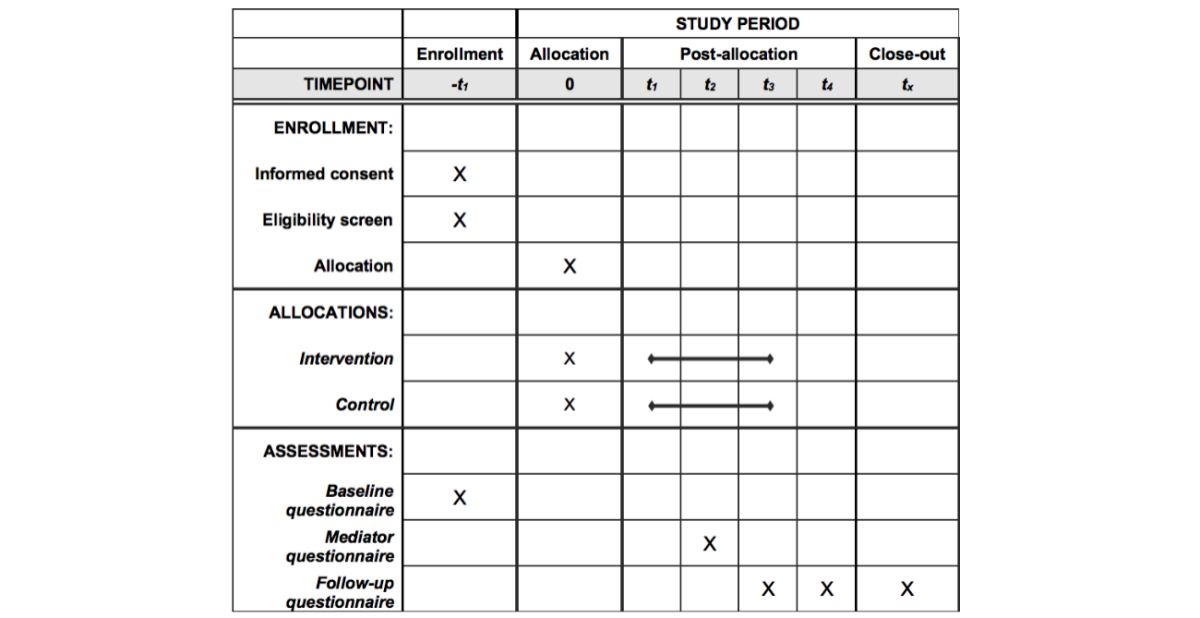
SPIRIT (Standard Protocol Items: Recommendations for Interventional Trials) checklist.

**Figure 2:**
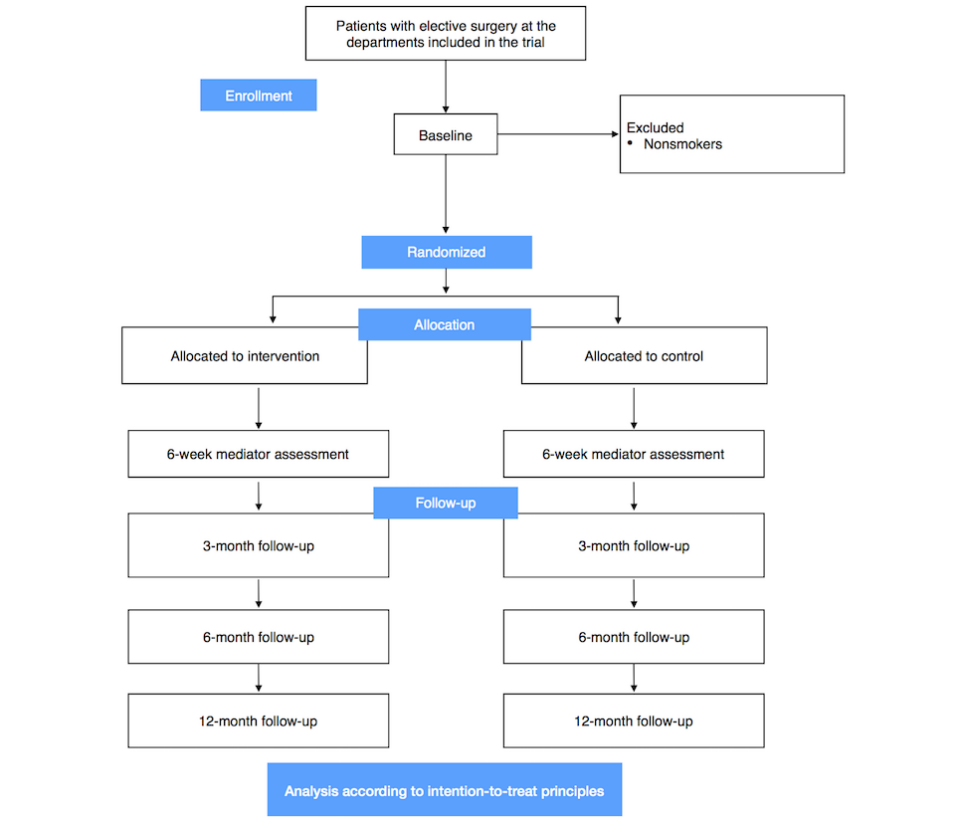
CONSORT (Consolidated Standards of Reporting Trials) flowchart.

To investigate the effect of the intervention, a randomized controlled trial (trial registration: ISRCTN33869008) will be conducted at 20 surgical departments in southeast Sweden. The trial will follow a two-arm single-blind parallel-group design. Figures 1 and 2 offer an overview of the design in the form of a SPIRIT (Standard Protocol Items: Recommendations for Interventional Trials) figure and CONSORT (Consolidated Standards of Reporting Trials) flowchart, respectively. The trial began in October 2018.

#### Intervention and Control Settings

One group will be given access to the novel intervention, while the other group will not be given access to the intervention. Both groups will have access to the surgical departments’ current routine for smoking cessation prior to surgery. The current routine varies slightly among departments, but generally consists of referring individuals to either the national quit-smoking helpline or their local primary health care provider. None of the participating departments’ supply any additional cessation help.

#### Recruitment

The study will be advertised through printed material provided to patients when they meet with a nurse or operation planner prior to surgery. During this meeting, the nurse or operation planner will emphasize on the importance of smoking cessation prior to surgery and hand out the printed material about the trial. Patients will also be informed about other support they can receive for smoking cessation.

Patients who want more information about the study will have to send an SMS message with a specific code to a dedicated phone number. In response, they will receive a message containing a hyperlink to a webpage that contains more information about the study. On the webpage, participants who wish to partake in the study will complete the informed consent form, following which a baseline questionnaire will be presented to them. Participants will be randomized once the baseline questionnaire has been completed.

Recruitment will be continuous for a minimum of 12 months and a maximum of 30 months. After the initial 12 months, the recruitment period will be extended in 6-month periods until a sufficient number of participants have been recruited according to power calculations (to a maximum of the 30 months).

This protocol was approved by the ethical review board in Linköping (DNR 2018/316-31).

#### Eligibility

Smokers undergoing elective surgery who own a mobile phone will be included in the study. No age restrictions will be applied. Patients will be excluded if they inform the operation planners that they do not smoke or if they report that they smoke zero cigarettes when filling out the baseline questionnaire.

#### Randomization

Randomization will be fully computerized, not employ any strata or blocks, and not be possible to subvert because all subsequent study processes are fully automated. The trial is single blind, as participants will be aware of group allocation. Since the randomization procedure is automated, group allocation will not be disclosed to individuals responsible for the research project (researchers and surgical staff).

#### Follow-Up

Six weeks after randomization, mediating factors will be assessed through a questionnaire. At 3, 6, and 12 months after randomization, smoking-cessation outcomes and mediating factors will be assessed through questionnaires. In all cases, hyperlinks to the questionnaires will be sent to participants’ mobile phones via an SMS. Two reminders will be sent, after which phone calls will be made to collect the primary outcome measures only. A maximum of five phone calls will be made to each participant at each follow-up stage.

#### Measures

All questions asked at baseline and subsequent follow-ups can be found in Multimedia Appendix 1.

Hypothesis 1:

- Primary outcome measures: Prolonged abstinence and point prevalence of smoking abstinence.
- Secondary outcome measures: Seven-day point prevalence of smoking abstinence, mean number of quit attempts since participation in the trial, number of uses of other smoking-cessation services since joining the trial, and number of cigarettes smoked weekly (if still smoking).

Hypothesis 2:

- Mediation measures: Importance and self-efficacy of quitting and staying smoke free.

Prolonged abstinence is defined according to the Russell standard definition [38], applying the usual threshold of not smoking more than 5 cigarettes in the past 8 weeks (thus allowing for a 4-week grace period). The 8-week period is then adjusted to 5 and 11 months at 6- and 12-months of follow-up, respectively. Point prevalence of smoking abstinence is defined as not smoking any cigarette in the past 4 weeks, which aims to capture delayed effects of the intervention, as suggested by the Society for Research on Nicotine and Tobacco [39]. Importance and self-efficacy will be investigated by two questions (Multimedia Appendix 1) rated on a scale of 1 to 10.

### Statistical Analysis

#### Methods

All analyses will be performed under the intention-to-treat principle, where all randomized individuals will be included. Missing outcome data will initially be handled using complete-case analysis, which assumes that data are missing at random. If data are systematically missing, it may be possible that early responders differ from late responders, and in extension, that late responders are more similar to nonresponders. We will therefore explore the plausibility of the missing-at-random assumption by regressing the primary outcomes on the number of follow-up attempts needed before a response was recorded. To further explore the missing-at-random assumption, attrition will be investigated among study groups by comparing baseline characteristics between those who did and those who did not respond at follow-up. A sensitivity analysis that includes imputed values for missing outcome data (using multiple imputation by chained equations) will also be performed. Data will be graphically examined for outliers or data input errors, and sensitivity analyses will be performed while excluding any erroneous data points.

#### Hypothesis 1

Primary outcome measures and 7-day point prevalence of smoking abstinence will be analyzed using logistic regression. Negative binomial regression will be used to analyze the number of quit attempts, other smoking cessation services used, and cigarettes smoked weekly. Both unadjusted and adjusted models (primary) will be investigated. The following baseline characteristics will be adjusted for all models: gender, age, years of smoking, mean number of cigarettes smoked weekly, use of snuff, Fagerströms Nicotine Dependence Scale [40], importance, and self-efficacy.

Effect-modification analyses will be performed for the two primary outcomes. The following potential effect modifiers measured at baseline will be explored: gender, age, years of smoking, mean number of cigarettes smoked weekly, use of snuff, Fagerström Nicotine Dependence Scale, importance, and self-efficacy. Each effect-modification analysis will be performed by comparing adjusted logistic regression models excluding and including the interaction parameter using the likelihood ratio test.

For all models, coefficients of interest will be assessed for statistical significance using a null hypothesis testing approach, where tests will be two-tailed at the .05 significance level with 95% CIs. Alongside the null hypothesis tests, posterior distributions using a Bayesian approach will be calculated for each coefficient [41]. Both significance tests and posterior distributions will create a basis for scientific inference.

#### Hypothesis 2

Mediators will be explored using a causal inference framework [42], where Monte Carlo methods are relied upon for inference. This allows for any type of model (linear and nonlinear) to be used to represent the relationships between the group allocation, mediating variable, and the outcome. Three models will be created for each outcome measure, two of which investigate the mediating factors on their own and a third that incorporates both mediators at once. If any baseline characteristics are found to moderate the effect in the primary analysis, additional mediator models will be created to include these characteristics as moderators.

#### Exploratory Analyses

As part of the primary investigator’s precision health initiative, we aim to include predictive modeling of the intervention. Traditionally, trials contrast the mean difference between two groups, but do not address individual variability. Intuitively, we know that some individuals will respond well to an intervention, others might not, and some might further be harmed by it. We aim to predict how individuals will respond to an intervention using only individual baseline characteristics. Therefore, we will measure characteristics at baseline related to the behavior-change theory, in particular, importance and self-efficacy, as well as conventional baseline characteristics (age, gender, etc). These characteristics will then be used to inform statistical models that predict individual outcomes.

Predictive analysis requires a radically different approach of assessing a model’s performance, as explaining and predicting are two different tasks [43]. We will use a Bayesian approach using shrinkage priors [44,45], which allows us to include all characteristics measured at baseline and learn which ones should be included in the predictive model from the data. The result is a model that can tell individuals how likely it is that the intervention has a positive effect on them specifically, rather than quoting the group mean difference.

### Power Calculation

A previous study that explored the effect of an SMS-based smoking-cessation intervention for Swedish university students found that 25.9% in the intervention group had not smoked more than 5 cigarettes in the past 8 weeks compared to 14.6% in the control group [16]. This is comparable to a Cohen *d* value of 0.3, which can be interpreted as a small-to-medium effect. In general, this is in line with what we should expect from brief digital lifestyle interventions. In order to identify a statistically significant difference between the two groups, under the expected Cohen *d* value of 0.3, we will need to recruit 192 patients per group (at 0.8 power and .05 significance level). Assuming a 10% loss to follow-up, the final sample size required is 434 patients.

With 20 surgical departments recruiting participants over 12 months, we would require each department to recruit an average of 1.8 participants each month. If necessary, the recruitment period of 30 months will require 0.7 recruited participants on an average per month per department. We believe that this is feasible, given the commitment from the departments.

## Results

At the time of submission of this protocol, recruitment had not yet started. Recruitment started in late October 2018 and is expected to last for a maximum of 30 months. The first results are expected to be available approximately 3 months after the final date of recruitment.

## Discussion

The project within which this trial is contained is focused on perioperative smoking cessation, mainly with a focus on reducing postoperative complications. However, more generally, smoking is responsible for approximately 9.6% of the total disease burden in Sweden, and the annual number of new younger smokers is between 16,000 and 20,000 [2,6]. Approximately 9% of the general population of Sweden comprises daily smokers [3]. Globally, smoking is the most important preventable cause of ill health and death, where for every death related to smoking, more than 20 additional individuals suffer from at least one serious smoking-related illness [1]. Thus, any opportunity presented to help individuals to quit smoking should be taken advantage of.

Surgical departments in Sweden are obligated to inform patients about complications related to smoking and surgery, but should also offer help to quit smoking. However, structural problems and scarcity of time and resources lead to patients simply being instructed to quit, and perhaps, referred to a primary health care clinic. Some surgical departments have staff dedicated to helping patients quit smoking, but not many departments can offer this resource. An SMS-based smoking-cessation aid can be effective in helping individuals quit smoking and is a very simple and time-efficient tool for surgical departments to use. Health care professionals only need to inform patients about a phone number, and patients can sign up by sending a single SMS message.

This trial will collect data on the effectiveness of the intervention and further the growing evidence on SMS-based interventions for smoking cessation. This will help decide whether the intervention should be made generally available for all surgical departments in Sweden. Furthermore, the participation of several departments in the trial will give us a unique opportunity to identify key barriers in current practice and adapt the sign-up procedure to reach as many patients as possible and disrupt current routines as little as possible. These insights will lay the foundation for future implementation of research projects. If the intervention is found to be effective, it might have a substantial impact on not only the number of postoperative complications but also the disease burden in Sweden caused by smoking, in general.

A limitation of this study is that is that it does not include cessation of smoke-free tobacco (such as snuff). To our knowledge, no studies have thus far identified an association between smoke-free tobacco and postoperative complications. Thus, the benefits of an intervention targeting smoke-free tobacco will have to be evaluated in a separate trial. Another limitation is the use of nonbiochemical verification at follow-up. However, the Society for Research on Nicotine and Tobacco recommends that in population-based studies with limited face-to-face contact, it is neither required nor desirable to use biochemical verification [39]. Finally, this trial has been designed to power the statistical analyses of two primary outcome measures; thus, analyses of secondary outcome measures and mediation measures should be regarded as preliminary and exploratory work.

## Appendix

Multimedia Appendix 1 Questionnaires.

## Abbreviations

SMS: short message service
SPIRIT: Standard Protocol Items: Recommendations for Interventional Trials
CONSORT: Consolidated Standards of Reporting Trials
