# Supplementary material for: Mobile Phone–Based Smoking-Cessation Intervention for Patients Undergoing Elective Surgery: Protocol for a Randomized Controlled Trial
Source: JMIR Res Protoc. 2019 Mar 26;8(3):e12511. doi: 10.2196/12511 (PMC6454332; doi:10.2196/12511)
Supplement: Multimedia Appendix 1 [file resprot_v8i3e12511_app1.pdf]

APPENDIX A – QUESTIONNAIRES

BASELINE QUESTIONNAIRE

1. Gender:
    a. Female
    b. Male
    c. Other
2. Age (numerical measure)
3. Do you know the date of your surgery?
    a. Yes
    b. No
    c. Unsure
4. In how many weeks will you undergo surgery? (numerical measure) (Only asked if a) to Question 3).
5. How many years have you smoked? (numerical measure)
6. How many cigarettes do you smoke per day/week/month? (numerical measure)
7. Do you use snus?
    a. No
    b. A few times each month
    c. A few times each week
    d. Daily – less than 1/3 box
    e. Daily – 1/3 box
    f. Daily – 1/2 box
    g. Daily – 1 box or more
8. How long after you have woken up do you smoke your first cigarette?
    a. Within 5 minutes
    b. After 6-30 minutes
    c. After 31-60 minutes
    d. After more than 60 minutes
    e. I don't smoke
9. Do you struggle to stay smoke free in situations where it is not allowed to smoke, e.g. at the cinema or during flights?
    a. Yes
    b. No
10. Which cigarette is hardest to stay away from?
    a. The one in the morning
    b. Any other cigarette
11. Do you smoke more during the mornings than during the rest of the day?
    a. Yes
    b. No
12. Do you smoke even if you are so ill that you are bedridden?
    a. Yes
    b. No
13. How many times have you tried to quit smoking? (numerical measure)
14. Have you ever received professional help, individually or in a group, to quit smoking?
    a. Yes, I am receiving help at the moment
    b. Yes, but not right now
    c. No

15. Have you ever called the quit smoking hotline (Sluta-Röka-Linjen)?
    a. Yes
    b. No
16. How important is it for you to quit smoking? (Scale measure between 1 and 10)
17. How confident are you that you will be able to quit smoking? (Scale measure between 1 and 10)

MEDIATION QUESTIONNAIRE

1. How important is it for you to quit smoking? (Scale measure between 1 and 10)
2. How confident are you that you will be able to quit smoking? (Scale measure between 1 and 10)

FOLLOW-UP QUESTIONNAIRE (3-, 6- AND 12-MONTHS)

1. Have you had your surgery?
    a. Yes
    b. No, but I know approximately when I will have it
    c. No, and I am not sure that the surgery will happen
2. How many weeks ago did you undergo surgery? (numerical measure) (Only asked if a) to Question 1)
3. In how many weeks do you think you will undergo surgery? (numerical measure) (Only asked if b) to Question1)
4. Have you smoked more than 5 cigarettes the past 8 weeks? (8 weeks will change to 5 months and 11 months at 6- and 12-month follow-up respectively).
    a. Yes
    b. No
5. Have you smoked any cigarette the past 4 weeks?
    a. Yes
    b. No
6. Have you smoked any cigarette the past 7 days? (Only asked if a) to Question 5)
    a. Yes
    b. No
7. How many cigarettes do you smoke per day/week/month? (numerical measure) (Only asked if a) to Question 6)
8. Do you use snus?
    a. No
    b. A few times each month
    c. A few times each week
    d. Daily – less than 1/3 box
    e. Daily – 1/3 box
    f. Daily – 1/2 box
    g. Daily – 1 box or more
9. How many quit attempts have you made since you joined the trial? (numerical measure)
10. Have you sought any other support to quit smoking since you joined the trial? (Multiple answers can be given) (Several options with available support mechanism, including hotline, prescription drugs, etc.)
11. How important is it for you to quit and stay smoke free? (Scale measure between 1 and 10)
12. How confident are you that you will be able to quit and stay smoke free? (Scale measure between 1 and 10)
